# Determinants of dietary diversity practice among pregnant women attending antenatal clinic at Wachemo University Nigist Eleni Mohammed memorial referral hospital, Southern Ethiopia

**DOI:** 10.1371/journal.pone.0250037

**Published:** 2021-04-09

**Authors:** Romedan Delil, Beakal Zinab, Hassen Mosa, Ritbano Ahmed, Habtamu Hassen

**Affiliations:** 1 Department of Clinical Nursing, Hossana College of Health Science, Hossana, Ethiopia; 2 Department of Nutrition and Dietetics, Institute of Health, Jimma University, Jimma, Ethiopia; 3 Department of Midwifery, College of Medicine and Health Sciences, Wachemo University, Hossana, Ethiopia; 4 Department of Medical Emergency, Hossana College of Health Science, Hossana, Ethiopia; Ordu University, TURKEY

## Abstract

**Background:**

Dietary diversity has continued to receive a global attention among pregnant women as they have been considered susceptible to malnutrition because of their increased nutrient demands. Thus, a variety of foodstuffs in their diet are necessary for ensuring the appropriateness of their nutrient consumptions. This study, therefore assessed the dietary diversity practice and its determinants among pregnant women attending antenatal clinic at Wachemo University Nigist Eleni Mohammed memorial referral hospital, Southern Ethiopia.

**Methods:**

A hospital-based cross-sectional study was carried out on 303 participants from May 1 to June 15, 2019 using a systematic random sampling technique. Data were entered and analyzed using SPSS (version24.0). Both bivariate and multivariable logistic regression analyses were used to decide the association of each explanatory variable with the outcome variable. Odds ratio with their 95% confidence intervals was calculated to identify the presence and strength of association, and statistical significance was declared at *p* < 0.05.

**Results:**

The overall prevalence of adequate dietary diversity practices was observed to be 42.6%. The determinants of dietary diversity practice included earning of a monthly income ≥2000 Ethiopian birr (AOR = 1.62; 95%CI:1.19–2.85), maternal educational level (AOR = 2.50; 95% CI: 1.05–6.12), educational status of partner (AOR = 2.45; 95% CI:1.20, 9.57), having a partner who was a government employee (AOR = 4; 95% CI:2.18–7.21), and the receiving of nutritional information (AOR = 1.35; 95% CI: 3.39–6.94).

**Conclusions:**

The study indicated that the overall consumption of adequate dietary diversity practice was found to be low. Therefore, increasing household income, enhancing nutritional related information, advancing the academic level of both wife and her partner is essential to improve women’s dietary diversity practice.

## Introduction

During the prenatal period appropriate nutrition is necessary to ensure the optimum outcomes of pregnancy and for the overall health of the mother [1]. More specifically children and pregnant mothers are the most vulnerable groups of the population for undernutrition due to inadequate intake of nutrition, low quality of diet, frequent infections, short inter-pregnancy intervals, food taboos, and poor food preparation [2].

In developing countries, micronutrient malnutrition remains a significant public health concern during pregnancy as it contributes to an increased risk of intrauterine growth restriction, low birth weight, preterm delivery, maternal morbidity and mortality [3, 4]. Also, dietetic insult during pregnancy might alter the structural and physiologic functional development of vital organs of fetal life and have a lifelong effect on later body constitution [5]. Pregnant women need a diet rich in micronutrients to supply the high demand of nutrients required for breast growth and uterus, and tissue synthesis. As a result, pregnant women are advised to eat a greater variety of food than usual [6].

Given that dietary diversity practice is a proxy indicator for nutrient appropriateness, it has continued to gain worldwide attention. Dietary diversity denotes an increased consumption food groups or diversity within food groups over a specific period that can promote optimal health, growth and development [7–9].

In developing countries women tend to have a monotonous diets that mainly comprise starch based foods lacking in vegetables, fruits and animal source foods, which leads to high level of malnutrition and micronutrient deficiencies [10, 11]. Studies indicate that the following were all associated factors in the dietary diversity practices of pregnant women: educational status, monthly income, dietary awareness, family size, age, possession of radio, having an illness, partner’s occupation, attending antenatal care (ANC), and receiving nutritional information [12–14]. Moreover, food misconceptions can adversely affect the dietary practices of pregnant women. These can include taboos about the consumption of green leafy vegetables, yogurt, cheese, sugar cane, green peppers, and other food items [15, 16].

In 2016, the Ethiopian government launched a national nutrition program and prioritized several interventions such as the promotion of maternal nutrition comprising the adequate intake of diversified foods to improve the nutritional status of the women [17]. However, the limited studies conducted in various towns of Ethiopia revealed that, the adequate dietary diversity practice of pregnant women in was insignificant, ranging from 19.9% in Gojjam [18] to 43% in Diredawa town [7]. In general, the importance of dietary diversity among pregnant women is well accepted since they have been considered more at risk to experience malnutrition. Thus, further exploration of the scope and potential contributing factors of dietary diversity practice is essential to improve maternal nutrition during pregnancy. Therefore, the purpose of this study was to assess dietary diversity practice and its determinants among pregnant women at Wachemo University Nigist Eleni Mohammed memorial referral hospital.

## Methods and materials

A hospital-based cross-sectional study was carried out from May 1 to June 15, 2019 among pregnant women attending the ANC clinic at Wachemo University Nigist Eleni Mohammed Memorial referral hospital (WUNEMMRH), in Hossana town, Hadiya Zone, Southern Nation’s Nationalities and Peoples Region at 232 kilometers south of Addis Ababa, the capital city of Ethiopia. WUNEMMRH gives preventive, curative and rehabilitative clinical services structured in four case teams in outpatient, inpatient, emergency and critical care, maternal, child health and obstetrics, and the operation theatre. The hospital was chosen due to its highest patient and client attendance. It is the biggest hospital in Hadiya Zone serving the Zonal population and bordering Zones and special woredas including Silte, Gurage, Halaba and Kambata-Tembaro.

The source population comprised all pregnant women attending the ANC clinic at WUNEMMRH during the study period, while the study population encompassed randomly selected pregnant women attending the ANC clinic at WUNEMMRH during the study period. Pregnant women who were critically ill or unable to communicate were excluded from the study.

The sample size of 306 was calculated using a single population proportion formula with the following considerations: the prevalence of dietary diversity practices is 25.4%, which was taken from the research conducted in Shashemane [19], with a 95% confidence interval, a margin error of 5%, and 5% non-response rate. A systematic random sampling technique was used to recruit the study participants. Since sampling interval was computed to be three, every third interval was used to enroll the participants.

The questionnaire was first prepared in English, translated into Amharic, and then translated back to English to ensure the consistency. Two different experts ensured consistency by translating it back to English. Data was collected in Amharic (which is the local language). Data was collected through a structured and pretested questionnaire used in face to face interviews. Dietary intake information was measured by asking participants to list all food items they consumed in the last 24 hours preceding the survey day. Midwives, four with diplomas and two with a bachelor’s degree, were recruited for the data collection and supervision. The data collectors and supervisors were given training on the content of the tool, the purpose of the study, and how to collect the data. The questionnaire was adapted from Food and Agriculture Organization (FAO) dietary diversity guidelines [20]. The validity of the questionnaire was approved through the proper application of validity criteria (content validity). Reliability was assured through stability (the instrument was given to the same respondents more than once under similar situations and it was found consistent).

Data were collected on socio-demographic data; obstetric related history, food groups and dietary diversity practice. The validity and reliability of the instrument was ensured in scientific manner. The questionnaire were pretested on 15 pregnant women in Hossana health center which is out of the study area before the actual data collection period. After the pretest, understand ability, clearness, and organization of the instrument was also checked. The supervisors and the investigators strictly monitored the data collection process every day over course of the pretest and the actual data collection period. Further, the filled tool was collected and signed by the supervisor after being observed for any lacking items and certainty.

### Measurement

For this study, a total of ten food groups was used and participants who consumed ≥5 food groups were categorized as having an adequate dietary diversity practice while those participants who consumed < 5 food groups was categorized as having an inadequate dietary diversity practice [20].

### Data processing and analysis

Data were entered and analyzed using SPSS (version 24.0). Descriptive statistics, frequency, and proportions were calculated to summarize the data. Logistic regression analyses were conducted to identify determinants of diversity dietary practice. Firstly, bivariate logistic regression analysis was conducted on all explanatory variables. Then, multivariable logistic regression was undertaken on variables that had a *p*-value ≤ 0.25 in the bivariate logistic regression analysis. The degree of association between explanatory variables, and outcome were evaluated using odds ratio with 95% confidence intervals. *P*<0.05 was stated as statistically significant. The Hosmer-Lemeshow statistic had a significance of 0.86 showing that the model was a good fit. Multi-collinearity was tested for interaction between explanatory variables through variance inflation factor which displayed a value of < 5.

### Ethics approval and consent to participate

Ethical clearance was obtained from the Institutional Review Board of Hossana College of Health Science. Formal letter was attained from the Hossana town health bureau. Then, permission was obtained from WUNEMMRH before starting the data collection. The participants were informed about purpose, procedures, potential risks and benefits of the study. Informed written consent was sought from selected participant to confirm their willingness to participate in the study before the interview. Parental or legal guardian consent was taken for respondents who were under 18 years of age. To keep confidentiality, name was not included in the written questionnaire. Additionally, the participants were ensured that refusal to consent or withdrawal from the study would not alter or put at risk their access to care.

## Results

### Socio demographic characteristics

From a total of 306 study participants, 303 were agreed to participate, making the response rate of 99%. The mean age of the participants was 28.3 ±2.7 years. Most of the participants, 294(97%) were married and 114(37.6%) had pursued an academic rank of college and above. Regarding mothers’ occupation, 194(64%) were housewives. With regard to income, 120(39.6%) of the participants earned a monthly income of 2000 and above Ethiopian Birr (ETB) (Table 1).

**Table 1:** Socio demographic characteristics of study participants in Wachemo University Nigist Eleni Mohammed Memorial referral hospital, 2019.

Variables	Frequency(n = 303)	Percent
**Age in year**		
<25	88	29.0
25–34	153	65.7
≥35	16	5.3
**Household income in ETB**		
<1000	90	29.7
1000–2000	93	30.7
≥2000	120	39.6
**Maternal education**		
No formal education	22	7.3
Primary education	59	19.5
Secondary education	108	35.6
College and above	114	37.6
**Educational status of husband**		
None	13	4.3
Primary	51	16.8
Secondary	92	30.4
College and above	147	48.5
**Religion**		
Orthodox	29	9.6
Protestant	255	84.2
Muslim	19	6.2
**Marital status**		
Married	294	97.0
Not married	9	3.0
**Maternal occupation**		
Housewife	194	64.0
Merchant	19	6.3
Government employee	83	27.4
Students	7	2.3
**Occupation of husband**		
Daily worker	86	28.4
Farmer	18	5.9
Merchant	154	50.8
Government employee	45	14.9
**Family size**		
<4	261	86.1
≥4	42	13.9

### Obstetrics related characteristics

Regarding ANC, nearly half of the participants, 155(51.2%) had three and more ANC visits and 192(63.4%) were multiparous. Concerning birth spacing, 109(46.2%) had a birth interval of ≤ 2 years and168 (55%) had not received any information about nutrition (Table 2).

**Table 2:** Obstetrics characteristics of study participants in Wachemo University Nigist Eleni Mohammed Memorial referral hospital, 2019.

Variables	Numbers(n = 303)	Percent
**Number of ANC visit**		
1–2	148	48.8
≥3	155	51.2
**Stage of pregnancy**		
First trimester	50	16.5
Second trimester	147	48.5
Third trimester	106	35.0
**Parity**		
Primipara	75	24.8
Multipara	192	63.4
Grand multipara	36	11.8
**Birth interval in year**		
≤2	109	36.0
3–4	129	42.6
≥5	65	21.5
**Age at marriage**		
<20	244	80.5
≥20	59	19.5
**Receiving nutritional information**		
Yes	135	44.6
No	168	55.4

In this study, the overall prevalence of adequate dietary diversity practices was observed to 129(42.6%). The most commonly consumed foods were cereals 298(98.3%), legumes 300(99%), and other vegetables 288(95%). However, eggs 129(42.5%) and animal source foods 153(50.5%) were the least consumed food groups (Table 3).

**Table 3:** Consumption of food groups and dietary diversity practice among study participants in Wachemo University Nigist Eleni Mohammed Memorial referral hospital, 2019.

Variables	Frequency	Percent
**Food groups**		
Egg	129	42.5
Other vitamin A rich fruit, tubers and vegetables	260	85.8
Cereals	298	98.3
Dark green leafy vegetables	260	85.8
Milk and milk product	181	59.7
Other fruits	262	86.4
Other vegetables	288	95
Meat, poultry and fish	153	50.5
Pulse or Legumes	300	99
**Dietary diversity practice**		
Adequate	129	42.6
Inadequate	174	57.4

### Determinants of dietary diversity practice

In this study after adjusted analysis, earning a monthly income of ≥ 2000 ETB, maternal and partner educational status, receiving nutritional information, and having a partner who was a government employee were determinants of dietary diversity practice.

The likelihood of having an adequate dietary diversity practices was1.62 times (AOR = 1.62; 95% CI; 1.19–2.85) higher among pregnant women whose monthly income was ≥2000 ETB compared to those who had a monthly income of <1000ETB.

Similarly, the odds of adequate dietary diversity practice was 2.5 times (AOR = 2.50; CI: 1.05–6.12) higher among pregnant women who had an educational level of college and above as compared to those who had no formal education.

Additionally, the likelihoods of having an adequate dietary diversity practices was higher among women having a partner who was a governmental employee and having a higher educational level as compared to their counterparts.

Furthermore, the odds of adequate dietary diversity practice was 1.35 times (AOR = 1.35; 95% CI: 3.39–6.94) higher among women who had received nutritional information as compared to their counterparts (Table 4).

**Table 4:** Determinants of dietary diversity practice among study participants in Wachemo University Nigist Eleni Mohammed Memorial referral hospital, 2019.

Variables	Dietary diversity practice		COR(95% CI)	AOR(95% CI)
	Adequate	Inadequate		
**Average monthly income in ETB**				
<1000	30 (33.3)	60 (66.7)	Reference	Reference
1000–2000	41 (44.1)	52 (55.9)	1.57(0.86–2.87)	1.50 (0.54–2.12)
> 2000	58 (48.3)	62 (51.7)	1.87(1.26–3.29)*	1.62(1.19–2.85)**
**Maternal educational status**				
No formal education	13 (59.1)	9 (40.9)	Reference	Reference
Primary education	35 (59.3)	24 (40.7)	0.99 (0.36–2.68)	0.75(0.31–2.23)
Secondary education	43 (39.8)	65 (60.2)	2.18(0.86–5.55)	1.92(0.78–4.85)
College and above	38 (33.3)	76 (66.7)	2.88(1.14–7.36)*	2.5(1.05–6.12)**
**Educational status of partner**				
No formal education	8 (61.5)	5 (38.5)	Reference	Reference
Primary education	24 (47.1)	27 (52.9)	1.80 (0.52–6.25)	1.91(0.76–7.32)
Secondary education	48 (52.2)	44 (47.8)	1.46 (0.45–4.82)	1.57(0.86–5.14)
College and Above	49 (33.3)	98 (66.7)	3.2(1.99–10.29)*	2.45(1.20–9.57)**
**Occupation of partner**				
Daily worker	25 (55.6)	20 (44.4)	Reference	Reference
Farmer	14 (77.8)	4 (22.2)	2.80(0.79–9.84)	2.25(0.63–8.90)
Merchant	70 (45.5)	84 (54.5)	2.80(0.79–9.84)	0.41(0.26–1.17)
Government employee	20 (23.3)	66 (76.7)	4.13(2.49–8.23)*	4(2.18–7.21)**
**Receiving nutritional information**				
No	77 (45.8)	91 (54.2)	Reference	Reference
Yes	52 (38.5)	83 (61.5)	1.36(4.46–7.17)*	1.35(3.39–6.94)**

* = p≤0.25

** = p < 0.05

## Discussion

The current study assessed the dietary diversity practice and its determinants among pregnant women attending antenatal clinic at Wachemo University Nigist Eleni Mohammed memorial referral hospital. In this study, the overall prevalence of adequate dietary diversity practice was observed to be 42.6%, which is comparable with the reported rates in Diredawa and Gondar, which were 43% and 40.1% [7, 21]. This similarity might be due to the resemblance of socio-demographic characteristics. However, this prevalence is considerably higher than the reported rates of 19.9%, 25.4%, and 19.6% in Gojjam, Shashemene and India, respectively [18, 19, 22]. The discrepancy could be attributed to the study area and to the implementation of various interventions within the study period. In contrast, this figure was lower than the reported rates found in America and Malaysia, which were 54 and 74%, respectively [23, 24]. The variation might be due to difference in study setting, cultural, food taboo, and available maternal health care services. Further, this variation could be an indicator of ineffectiveness of the national strategies for maternal food and nutritional policies.

In this study, maternal and partners educational level were determinants with dietary diversity practice.This finding is consistent with studies carried out in Shashemene [19], Gondar [21], and Kenya [25]. This might be due to the fact that educated women and their partners have better understanding about the importance of consuming diversified foods. In addition, educated women can have a better job opportunity and income which can further advance their home food security status and intake of diversified diets.

This study also showed that higher level of household income was determinant of dietary diversity practice, which is in line with the studies conducted in Shashemene [19], Gondar [21] and Kenya [25]. The reason could be that households with a greater income will have a better access to consume various foodstuffs. Consequently, the dietary diversity practice of pregnant women in this household will be boosted.

The current study also revealed that occupation of partner was found to have a significant association with dietary diversity practice. This finding is consistent with the conduced in Kenya [25]. This might be because of women whose husbands are government employee have a better chance of earning salary and therefore a better access to diversified foods.

Furthermore, receiving nutritional information was determinant of dietary diversity practice, consistent with the studies conducted in Gondar [21] and north east Ethiopia [26]. This could be explained by the fact that women who received information about nutrition are more expected to consume diversified food than those women who do not received nutritional information. This could be explained by the fact that women who received information about nutrition will have a better awareness and comprehension to eat diversified foodstuffs than those women who do not received nutritional information.

This study has some limitations that should be addressed in the paper. In this regard, the result of the study might not be generalized to the general population since the study was conducted in the hospital. Also, this study was prone to recall bias. Besides, our study adopted a quantitative method alone. To balance out these limitations, dietary diversity practicewas measured by a validated and standardized instrument which was pretested and revised. Additionally, the statistical analysis methods used in this study was appropriate and effective to determine an associations between the outcome and independent variables.

## Conclusion

The obtained results shown that, the overall consumption of adequate dietary diversity was found to be low in the study area. The determinants of dietary diversity practice included earning a monthly income of ≥ 2000 ETB, receiving of nutritional information, having a partner who was a governmental employee, maternal and partner’s educational status. Therefore, increasing household income, enhancing nutritional related information, advancing the academic level of both wife and her partner are essential to improve women’s dietary diversity practice. Further studies using qualitative method should be conducted for better exploring the women’s voices and opinions.

## Supporting information

S1 File Questionnaire. (DOC)

S2 File SPSS. (SAV)

## Abbreviations

ANC: Antenatal care
AOR: Adjusted Odds Ratio
COR: Crude Odds Ratio
ETB: Ethiopian birr
FAO: Food and Agriculture Organization
SPSS: Statistical Package for Social Sciences
WUNEMMRH: Wachemo University Nigist Eleni Mohammed Memorial Referral Hospital

## References

[1] Saaka M. Maternal dietary diversity and infant outcome of pregnant women in Northern Ghana. International Journal of Child Health and Nutrition. 2013; 1(2):148–56.

[2] Harris-Fry H, Azad K, Kuddus A, Shaha S, Nahar B and Hossen M. Socio-economic determinants of household food security and women’s dietary diversity in rural Bangladesh: A cross-sectional study. Journal of Health Population and Nutrition. 2015; 33:1–12. 10.1186/s41043-015-0022-0 26825273 PMC5026026

[3] Abu-Saad K and Fraser D. Maternal nutrition and birth outcomes. Epidemiol Reviews. 2010; 32:21–5. 10.1093/epirev/mxq001 20237078

[4] Haider BA, Yakoob MY and Bhutta ZA. “Multiple-micronutrient supplementation for women during pregnancy,” Cochrane Database of Systematic Reviews. 2017; 13(4). 10.1002/14651858.CD004905.pub5 28407219 PMC6478115

[5] Gluckman PD, Hanson MA and Cooper C. In utero and early-life conditions and adult health and disease. The New England Journal of Medicine. 2008; 359(14):1524. 18832254 10.1056/NEJMc081629

[6] Nana A and Zema T. Dietary practices and associated factors during pregnancy in northwestern Ethiopia. BMC Pregnancy and Childbirth. 2018; 18:183. Available at 10.1186/s12884-018-1822-1. 29801471 PMC5970492

[7] Shenka A, Damena. M, Abdo. M and Roba. K. Dietary Diversity and Nutritional Status of Pregnant Women Attending Public Hospitals in Dire Dawa City Administration, Eastern Ethiopia. East African Journal of Health and Biomedical Sciences.2018; 2 (1): 10–17.

[8] Arimond M, Wiesmann D, Becquey E, Carriquiry A, Daniels MC and Deitchler M. et al. Simple food group diversity indicators predict micronutrient adequacy of women’s diets in 5 diverse, resource-poor settings. Journal of Nutrition. 2010; 140:2059S–69S. 10.3945/jn.110.123414 20881077 PMC2955880

[9] Kennedy G, Ballard T and Dop MC. Guidelines for measuring household and individual dietary diversity. Rome (Italy): Food and Agriculture Organization of the United Nations. 2010. http://www.fao.org/3/a-i1983e.pdf.

[10] Darnton-Hill I, Mkparu UC. Micronutrients in pregnancy in low- and middle-income countries. Nutrients. 2015; 7:1744–68. 10.3390/nu7031744 25763532 PMC4377879

[11] Lee SE, Talegawkar SA, Merialdi M and Caulfield LE. Dietary intakes of women during pregnancy in low and middle-income countries. Public Health Nutrition. 2013; 16(8):1340–53. 10.1017/S1368980012004417 23046556 PMC10271363

[12] Tafera B, Mideksa S and Dida N. Assessment of dietary practice and associated factors among pregnant mother in Ambo District, West Shoa, Oromia, Ethiopia. Ethiopian Journal Reproductive Health. 2018; 10(4):43–51.

[13] Gemeda D, Fekadu B, Wondu G and Habitamu F. Assessment of nutritional practice of pregnant mothers on maternal nutrition and associated factors in Guta Gida Woreda, East Wollega Zone, Ethiopia. Star Journal. 2013; 2(3):105–13.

[14] Tenaw Z, Arega M and Tachbele E. Nutritional knowledge, attitude and practices among pregnant women who attend antenatal care at public hospitals of Addis Ababa, Ethiopia. International Journal of Nursing and Midwifery. 2018; 10(7):81–9.

[15] Taruvinga A, Muchenje V and Mushunje A. Determinants of rural household dietary diversity: the case of Amatole and nyandeni districts, South Africa. International Journal of Development and Sustainability. 2013; 2(4):2233–47.

[16] Zerfu TA, Umeta M and Baye K. Dietary habits, food taboos, and perceptions towards weight gain during pregnancy in Arsi, rural central Ethiopia: a qualitative cross-sectional study. Journal of Health and Population Nutrition. 2016; 35:22. Available at: 10.1186/s41043-016-0059-8. PMC5025964 27456151

[17] Ethiopia, F.D.R.E. National nutrition program. 2016–2017.

[18] Demilew YM, Alene GD, Belachew T. Dietary practices and associated factors among pregnant women in West Gojjam Zone, Northwest Ethiopia. BMC Pregnancy and Childbirth. 2020;20(18):1–11. Available at: 10.1186/s12884-019-2702-z. 31906981 PMC6945405

[19] Desta M, Akibu M, Tadese M, Tesfaye M. Dietary Diversity and Associated Factors among Pregnant Women Attending Antenatal Clinic in Shashemane, Oromia, Central Ethiopia: A Cross-Sectional Study. HindawiJournal of Nutrition and Metabolism. 2019;7–10. Available at: 10.1155/2019/3916864. PMC6434279 30993019

[20] Food and Agricultural Organization, Minimum Dietary Diversity for Women’s; A Guidelines to Measurement, Food and Agriculture Organization of the United Nations, FANTA, Rome, Italy. 2016.

[21] Alemayehu MS and Tesema EM. Dietary practice and associated factors among pregnant women in Gondar town North West, Ethiopia, 2014. International Journal of Nutrition and Food Science. 2015; 4(6):707–12. Available at: 10.11648/j.ijnfs.20150406.27.

[22] Willy K, Judith K, and Peter C. Dietary Diversity, Nutrient Intake and Nutritional Status among Pregnant Women in Laikipia County, Kenya. International Journal of Health Sciences & Research, 2016; 6(4):378–385.

[23] Shehab L. Nutritional Awareness of Women during Pregnancy. Journal of American Science. 2012; 8(7).

[24] Mirsanjari M, Ahmad A, Shukri M and Mosavat M. Does Nutritional Knowledge Have Relationship with Healthy Dietary Attitude and Practices during Pregnancy? International Conference on Nutrition and Food Sciences, Singapore. 2012; 39.

[25] Shashikantha SK, Sheethal MP and Vishma BK. Dietary diversity among women in the reproductive age group in a rural field practice area of a medical college in Mandya district, Karnataka, India. International Journal of Community Medicine and Public Health. 2016;3(3):746–749. Available at: 10.18203/23946040.ijcmph20160644.

[26] Aliwo S, Fentie M, Awoke T and Gizaw Z. Dietary diversity practice and associated factors among pregnant women in North East Ethiopia. BMC Research Notes. 2019; 12:123. Available at: 10.1186/s13104-019-4159-6. 30845950 PMC6407270

