# Supplementary material for: Determinants of dietary diversity practice among pregnant women attending antenatal clinic at Wachemo University Nigist Eleni Mohammed memorial referral hospital, Southern Ethiopia
Source: PLoS One. 2021 Apr 9;16(4):e0250037. doi: 10.1371/journal.pone.0250037 (PMC8034717; doi:10.1371/journal.pone.0250037)
Supplement: S1 File — (DOC) [file pone.0250037.s001.doc]

**ANNEXE: I. English Version Questionnaire**

| Participant Identification: Address: ________________Kebele; ______________ Gote______________.  Client Telephone_________________. Code number____________. |
| --- |
| Date of interview (dd/mm/yyyy)____||____|____| Name of health facility_______________________ |

SECTION-1: Socio-economic and Demographic Characteristics

| S.No | Questions | Coding categories |
| --- | --- | --- |
| 101 | How old are you? Year of Birth? | _____________year |
| 102 | What is your current marital status? | 1. Single 2. Married 3. Divorced  4. Widowed 5. Separated 6. Other (specify)_______ |
| 103 | Ethnicity | 1. Hadiya 2. Kambata 3. Siliti  4. Amhara 5. Gurage 6. Others (specify)________ |
| 104 | What is your Religion? | 1. Orthodox 2. Protestant 3. Catholic  4. Muslim 5. Others (specify)_________ |
| 105 | Educational level of mother? | 1. Can’t read & write 2. Grade 1-4 3. Grade 5-8 4. Grade 9-10 5. Grade 11-12 6. College/university |
| 106 | Education status of partner | 1. Can’t read & write 2. Grade 1-4 3. Grade 5-8 4. Grade 9-10 5. Grade 11-12 6.College/university |
| 107 | Occupation of mother | 1. House wife 2. Merchant 3. Daily worker 4. Government employee 5. Other specify_________ |
| 108 | Occupation of partner | 1. Government employee 2. Farmer 3. Merchant  4. Daily worker 5. Other specify___________ |
| 109 | Number of people live in your household? | _______________. |
| 110 | Household monthly income (Probe all sources of income) | |___|___|___|___|___|___| ( write one digit in each parenthesis) |

***SECTION: 2.*** Pregnancy and Health Related Maternal Factors

| S. No | Questions | Coding categories |
| --- | --- | --- |
| 111 | Age at marriage? | ___________year |
| 112 | Age at first birth? | ___________year |
| 113 | Total number of pregnancy including the current one? | _____.if Primigravida 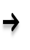120 |
| 114 | How many of the pregnancies were given still birth? | ____________. |
| 115 | Do you have an experience of abortion? | 1. Yes 2. No |
| 116 | If yes question 115, how many times? | ____________. |
| 117 | How many months are there b/n the previous and the current pregnancy? | ____________. |
| 118 | If she is not primigravida, history of ANC at previous pregnancy? | 1. Yes 2. No |
| 119 | If she is not primigravida, place of delivery for previous pregnancy? | 1. Home 2. Health facility |
| 120 | Last normal menstrual period of the women, gestational age in week? | ___________weeks. |
| 121 | How many times did you visit antenatal care service with this pregnancy? | ____________. |
| 122 | Have you ever taken Health education about Nutrition during pregnancy? | 1. Yes 2. No |
| 123 | If yes question 133, what kinds of information did you get? | 1. Iron source food  2. Additional meals  3. Healthy diets |
| 124 | Do you have garden in your home? | ________________________. |

***SECTION: 3.* Nutrition related and Dietary Intake Factors**

3.1. Maternal 24 Hours Dietary Recall

| Steps | Multiple pass 24 hours | Food eaten | Remark |
| --- | --- | --- | --- |
| First pass | Quick List |  |  |
| Second pass | Detailed description |  |  |
| Third pass | Review |  |  |

Please describe the foods (meals and snacks) that you ate yesterday during the day and night, whether at home or outside the home; Start with the first food eaten in the morning (Sunrise time yesterday to Sunrise today), Write down all food and drinks mentioned by the respondent.

| Meal | Breakfast | Snack | Lunch | Snack | Dinner | Snack | Remark |
| --- | --- | --- | --- | --- | --- | --- | --- |
| Food items  (Eaten with in the last 24hrs) |  |  |  |  |  |  |  |

When the respondent recall is complete, fill in the food groups based on the information recorded above, for any food groups not mentioned.

3.2. Food Frequency Questionnaire

| S. No | Question | Possible alternatives | Remark |
| --- | --- | --- | --- |
| 125 | How many meals per day do you eat on current pregnancy? | 1. Once 2. 2 times 3. 3times  4. 4 times 5. ≥5 times 6. I don’t know |  |
| 126 | Do you take milk &milk product? | 1. Yes 2. No | 2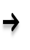128 |
| 127 | What types of dairy, milk and milk products do you take? | 1. Whole milk 2. Cheese 3. Powdered milk  4. Yoghurt 5. Butter milk 6. Low fat milk 7.Butter |  |
| 128 | Do you eat egg? | 1. Yes 2. No | 2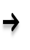130 |
| 129 | What types of egg do you eat? | Hen, duck or any other egg |  |
| 130 | Do you eat cereals& ready-to-eat? | 1. Yes 2. No | 2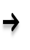133 |
| 131 | Do you eat white tubers & roots? | 1. Yes 2. No |  |
| 132 | What types of Cereals, white tubers and roots or Starchy staples do you eat? | A. Cereals and grains including ready-to-eat;  1. Wheat (such as whole meal/white bread)  2. Barley 3. Oats/aja 4. Rice 5. Maize  6. Spaghetti/pasta 7. Macaroni 8. Sorghum  9. Teff 10. Other (millet, sago, semolina, triticale)  B. White tubers and roots; potatoes, yams, cassava (White), godere, boyna or other foods made from roots |  |
| 133 | Do you eat Dark Green Leafy Vegetables? | 1. Yes 2. No | 2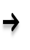135 |
| 134 | What types of Dark Green Leafy Vegetables do you eat? | 1. Kale 2. Swiss chard 3. Endive 4. Lettuce  5. Pumpkin leaves 6. Spinach 7. Cassava leaves  8. Mustard greens 9. Broccoli 10. Others________. |  |
| 135 | Do you eat ripe mangoes, papaya, bananas and peaches (dried, raw)? | 1. Yes 2. No | 2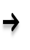137 |
| 136 | What types of other Vitamin A rich Fruits, Tubers and Vegetables do you eat? | Mango, papaya (Ripe, fresh and dried), pumpkin, carrots, sweet potatoes, red sweet pepper, red palm fruit/pulp, passion fruit, melon, deep yellow or orange-fleshed bananas, peaches (dried, raw) |  |
| 137 | Do you eat other fruits and vegetables? | 1. Other fruits; 1. Yes 2. No  2. Other vegetables; 1. Yes 2. No | 2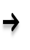139 |
| 138 | What types of other fruits and Vegetables do you eat? | 1. Fruits items; avocado, apple, mandarin orange, Dates, white/cream-fleshed bananas, strawberry, Grapes, Pears, not ripe mango or papaya, orange, lemon, pineapple, cherries, olive, peach, guava, citron, casimire, Wild fruits  2. Vegetables items; Beet root, common cabbage, onion, green beans, Cucumber, tomatoes, peas (fresh), green pepper, cauliflower, garlic, mushroom, chillies, green maize, leek, beets, ginger, wild vegetables. |  |
| 139 | Do you eat meat, poultry and fish? | 1. Yes 2. No | 2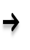141 |
| 140 | What types of organ meat, meat and poultry, and fish/seafood do you eat? | 1. Liver, kidney, heart or all other organ meats like tripe 2. Lamb, mutton, beef, veal, goat 3. Poultry (e.g. chicken, guinea fowl) 4. Fresh or dried fish, seafood |  |
| 141 | Do you take Nuts and seeds? | 1. Yes 2. No | 2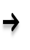143 |
| 142 | What types of Nuts and seeds do you eat? | 1. Almond 2. Sunflower 3. Linseed 4. Melon seeds 5. Sesame 6. ፈጦ 7. Other specify________. |  |
| 143 | Do you take Pulses or legumes? | 1. Yes 2. No | 2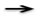145 |
| 144 | What types of pulses or legumes do you eat? | 1. Peas 2. Chickpeas 3. Horse bean 4. Lentils  5. Vetch 6. Haricot beans 7. Kidney bean  8. Niger seed 9. Fenugreek 10. Other specify______ |  |
| 145 | Do you eat products of Enset? | 1. Yes 2. No |  |
| 146 | What items of Enset do you eat? | Atakano, bulla, kocho, amicho |  |
| 147 | Women dietary diversity practice (based on above food group taken) | ___________________________food groups |  |
